# Two Years of Darkness: An Autobiographical Case Report of an Emergency Physician With Bilateral Retinal Detachments

**DOI:** 10.7759/cureus.22873

**Published:** 2022-03-05

**Authors:** Camiron Pfennig

**Affiliations:** 1 Emergency Department, Prisma Health/University of South Carolina School of Medicine Greenville, Greenville, USA

**Keywords:** giant retinal tear, posterior vitreous detachment, scleral lenses, vitrectomy, keratoconus, retinal detachment

## Abstract

Retinal detachments constitute an emergency ocular condition when the neurosensory retina separates from the retinal pigment epithelium, leading to the death of the tissue. Prompt diagnosis and treatment are essential to avoid significant morbidity, including vision loss and/or blindness associated with this condition. This case report describes the author’s challenging journey from a non-ophthalmologist perspective through the terrifying experience of bilateral rhegmatogenous retinal detachments involving seven surgical procedures prior to return to full clinical function.

## Introduction

Retinal detachments constitute an emergency ocular condition when the neurosensory retina separates from the retinal pigment epithelium (RPE), leading to the death of the tissue. Prompt diagnosis and treatment are essential to avoid significant morbidity, including vision loss or blindness associated with this condition [1]. Specifically, a rhegmatogenous retinal detachment (RDD) occurs when a tear, break, or hole occurs in the retina. When a break occurs, this allows the vitreous to enter the space underneath the retina, causing a detachment from the RPE. The incidence of RRD varies between studies from one in 10,000 to other studies, showing the annual risk to be between 6.3 and 17.9 per 100,000 [2]. The other eye in patients with RRD is at high risk of developing bilateral RRD with a prevalence of 7% [3].

This case report describes the author’s challenging journey from the perspective of a non-ophthalmologist through the terrifying experience of bilateral retinal detachments spaced two years apart involving seven surgical procedures prior to return to full clinical function. The article focuses on the presentation, the diagnosis, the treatment, and ultimately the return to practicing emergency medicine. My goal in writing this autobiographical case report is to provide a resource for my colleagues regarding the importance of the rapid diagnosis and honest discussion on the prognosis required for retinal detachment management.

## Case presentation

I was the unlucky little girl walking around with coke-bottle-thick glasses in first grade. Ultimately, I was diagnosed with bilateral keratoconus while in junior high and was no stranger to the optometrist, transitioning from glasses, to soft contacts, to rigid-gas permeable contacts, and ultimately to scleral lenses prior to completing my emergency medicine residency. Without my scleral lenses, I am legally blind. In addition to my many optometry visits, I had occasional corneal ophthalmology evaluations, including the placement of intrastromal corneal ring segments (Intacs) in an attempt to flatten the cornea and prevent the need for corneal transplants.

**Figure 1:**
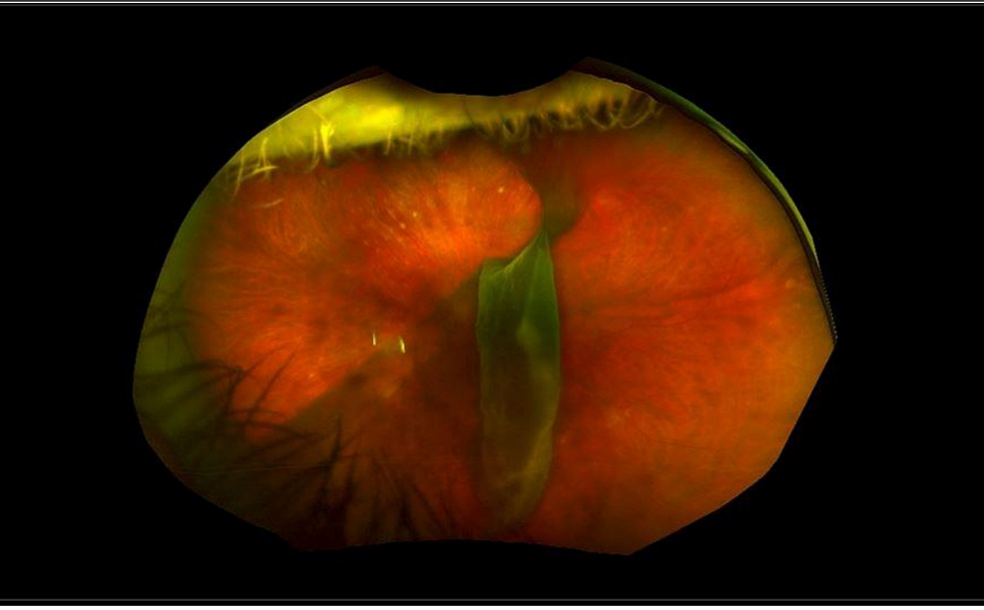
Left Retinal Tear

However, I never imagined that I would become a regular at the retinal specialist until that scary night on October 14th, 2018. Ten days prior to that memorable night, my one-year-old daughter accidentally hit me in the left eye on the airplane on the way home from the annual American College of Emergency Physicians’ (ACEP) Scientific Assembly. I began experiencing floaters and was initially diagnosed with a vitreous detachment upon return home. I was told that the floaters would worsen before they got better. When I started to experience the ominous "black curtain" sign, I blamed it on an expanding floater, the normal progression. On that cold Sunday evening before a scheduled overnight shift, I started panicking, realizing that I had a retinal detachment that I had been avoiding. Unfortunately, my vitreous detachment pulled the entire retina away, and I was ultimately diagnosed with a large retinal detachment with concern that the macula may be off. At that moment, I realized that I may only have vision out of one eye for the rest of my life. The images of my large left retinal tear prior to my surgery are shown in Figure 1.

**Figure 2:**
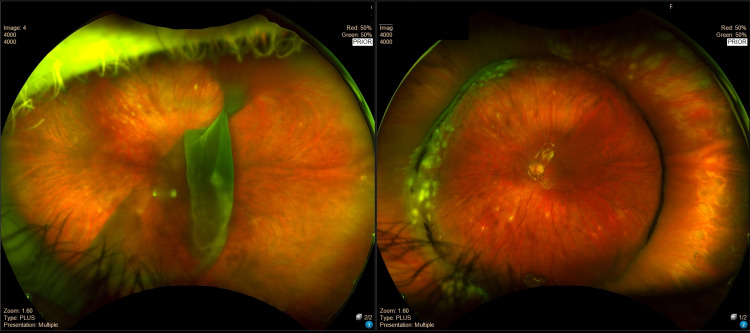
Left Retinal Tear Before and After Surgical Intervention

I was urgently rushed to the operating room (OR) for vitrectomy surgery, including laser treatment, gas bubble, and scleral buckle due to the extremely large tear. Fortunately, the macula was still attached and there was hope for a meaningful return of my eyesight. The recovery process included tortuous days and nights lying face down with an eye patch and a plethora of eye drops. Two months later, following the initial surgery, I had to return to the OR to have the gas bubble removed. Even though the retina had been repaired, I still was unable to go back to my scleral lenses until healing was completed. This left me with vision completely dependent on my right eye for three more months. The images of my large retinal tear in the left eye prior to my surgery next to my repaired retina following surgery are shown in Figure 2.

I was blessed to become pregnant in 2019 but, during my pregnancy, I developed a large left cataract secondary to the gas bubble disrupting the metabolism of the crystalline lens, leading again to near-complete vision loss secondary to the opacity of the cataract. However, I was unable to find a physician who would perform this elective procedure under the required general anesthesia while I was pregnant leading to the continued worsening of the cataract. Things became even more complicated when I had yet another surgery, but this time, it would be a cesarean section due to the theoretical risk of retinal detachment during spontaneous vaginal delivery.

Due to the complexities expected with the cataract removal, I was referred out of state to a specialist. However, the timing of the cataract surgery aligned perfectly with the start of the coronavirus disease 2019 (COVID-19) pandemic, and combined with travel restrictions and surgery postponements, I suffered from monocular vision again for another six months. At this point, I had been with monocular vision for a total of eleven months. Everything went well with the cataract repair, and I was eagerly awaiting healing so I could again get fitted for a new scleral lens and move forward with binocular vision.

**Figure 3:**
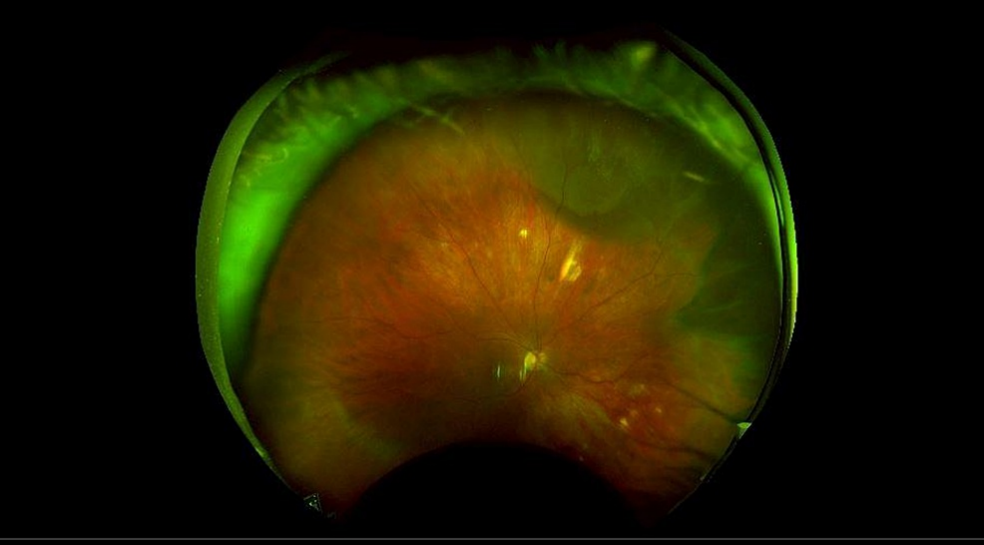
Right Retinal Detachment

Unfortunately, it was a cold day in October of 2020 when I spontaneously developed floaters in my right eye, which at the time was my only functional eye, while working an emergency department shift. I was again diagnosed with a vitreous detachment after a dilated exam and a reassuring ultrasound. However, I can tell you exactly the date and time that the retina detached after my horrific experience with my previous detachment. It was one of the scariest moments of my life, as I essentially became completely blind in less than a minutes' time as the curtain moved over my right eye and the cataract obstructed all vision in my left eye. The right retinal detachment is shown in Figure 3. I couldn’t see my patients, I couldn’t see my children, and at that point, I couldn’t see my future. I was devastated and at that time, I felt my career as an emergency physician was in severe jeopardy.

I was urgently taken to the OR once again. Fortunately, there was time in pre-op to pump to provide nutrition for my infant prior to more medication and more anesthesia. My right retinal detachment was repaired successfully, but the healing process would again be very long and very lonely considering I could not see out of either eye for another three months.

The left eye was not healing as expected after the cataract removal and a YAG (yttrium aluminum garnet) laser capsulotomy had to be performed while the right eye was in the early stages of vitrectomy recovery. Following the YAG procedure, I was able to get refit for a very expensive, custom-made scleral lens in the left eye intricately constructed to restore the vision in my left eye.

History repeated itself when, following the right eye vitrectomy, I then again developed a dense cataract requiring removal and healing prior to getting refitted for another scleral lens.

There have been many bumps in the road in the recovery process in both eyes, including recurrent iridocyclitis, but my calendar is now only sporadically occupied with visits to the corneal specialist, the retinal specialist, and the scleral optometrist. I owe my vision, my career, and ultimately my life to the amazing teams in Nashville, Tennessee, Atlanta, Georgia, and Greenville, South Carolina. With the intricate work and incredible talent of a team of ophthalmologists and optometrists, I have successfully returned to clinical duty, and it is a true miracle that I can see almost 20/20 bilaterally with my scleral lenses.

## Discussion

Rhegmatogenous retinal detachments (RRD) are the most common type of detachments and are caused by fluid passing from the vitreous cavity via a retinal tear or a break into the potential space between the sensory retina and the retinal pigment epithelium. Vision is potentially recoverable if the macula remains attached, and the retina gets appropriately reattached. However, if the macula comes off, vision may remain diminished despite surgical intervention. As a patient diagnosed with severe myopia and keratoconus at a young age, I was predisposed to breaks or tears in the neurosensory retina that could lead to an RRD [4]. When combined with a trauma, my risk for retinal detachment skyrocketed. In fact, it has been estimated that blunt trauma alone accounts for 10-20% of all retinal detachments [5].

Keratoconus is a bilateral progressive corneal ectasia characterized by progressive corneal steepening and thinning, induced myopia, and astigmatism. Estimates for the prevalence of keratoconus have varied historically, with a reported prevalence of around 0.05% [6]. Keratoconus should be considered in younger patients with myopia and astigmatism requiring frequent prescription changes as early diagnosis and treatment can be vital for preventing severe loss of vision.

In both scenarios, I was first diagnosed with a posterior vitreous detachment (PVD) when a portion of the vitreous gel lining the retina peeled away from the retina. A PVD is harmless by itself, though the floaters are often very distracting. Unfortunately, 10-15% of patients with symptomatic posterior vitreous detachment will develop a retinal defect, as the vitreous pulls away from the retina, especially in the periphery where the retina is thinner [7]. The risk of a retinal tear from a PVD is highest during the first four to six weeks after the initial symptoms occur, making it extremely important to educate patients with a PVD that they must seek emergent care if they begin to have large new floaters or increasing flashes of light, as these are ominous signs of a retinal tear or detachment. Ten days after my left PVD was diagnosed, I experienced severe visual field loss as a dark shadow appeared in my peripheral vision that progressed centrally within a few hours [8].

The expert retinal specialist ultimately decided to repair my first retinal detachment on the left with scleral buckling, vitrectomy, and intravitreal gas injection due to the location and size of the defect. My second detachment on the right was able to be repaired by scleral buckling, vitrectomy, and air tamponade rather than gas, to avoid yet another procedure to remove the gas. With primary vitrectomy, there is a good success rate in more complicated forms of RRD, but the major drawback of the procedure is the high incidence of postoperative cataract formation [9-10].

## Conclusions

My challenging journey through the terrifying experience of bilateral rhegmatogenous retinal detachments involving seven surgical procedures had a reassuring outcome thanks to the dedicated and talented corneal specialists, the retinal specialists, and the scleral optometrists. It is crucial to recognize the risk factors associated with retinal detachment and understand the importance of the rapid diagnosis and honest discussion on the prognosis required for retinal detachment management.

## References

[1] Mitry D, Charteris DG, Fleck BW, Campbell H, Singh J (2010). The epidemiology of rhegmatogenous retinal detachment: geographical variation and clinical associations. Br J Ophthalmol.

[2] Mitry D, Singh J, Yorston D (2012). The fellow eye in retinal detachment: findings from the Scottish Retinal Detachment Study. Br J Ophthalmol.

[3] Johnston T, Chandra A, Hewitt AW (2016). Current understanding of the genetic architecture of rhegmatogenous retinal detachment. Ophthalmic Genet.

[4] Liu X, Wang L, Wang C, Sun G, Liu S, Fan Y (2013). Mechanism of traumatic retinal detachment in blunt impact: a finite element study. J Biomech.

[5] Bak-Nielsen S, Ramlau-Hansen CH, Ivarsen A, Plana-Ripoll O, Hjortdal J (2019). Incidence and prevalence of keratoconus in Denmark - an update. Acta Ophthalmol.

[6] Hikichi T, Trempe CL (1994). Relationship between floaters, light flashes, or both, and complications of posterior vitreous detachment. Am J Ophthalmol.

[7] Gariano RF, Kim CH (2004). Evaluation and management of suspected retinal detachment. Am Fam Physician.

[8] Heimann H, Bornfeld N, Friedrichs W, Helbig H, Kellner U, Korra A, Foerster MH (1996). Primary vitrectomy without scleral buckling for rhegmatogenous retinal detachment. Graefes Arch Clin Exp Ophthalmol.

[9] Ryan EH, Joseph DP, Ryan CM (2020). Primary retinal detachment outcomes study: methodology and overall outcomes—primary retinal detachment outcomes study report number 1. Ophthalmol Retina.

[10] Sultan ZN, Agorogiannis EI, Iannetta D, Steel D, Sandinha T (2020). Rhegmatogenous retinal detachment: a review of current practice in diagnosis and management. BMJ Open Ophthalmol.

